# To Remove Rust from Instruments

**Published:** 1896-12

**Authors:** 


To Remove Rust from Instruments. In order to remove rust from instruments Sänger (*Berl. klin. Wochenschrift*, No. 26) recommends the following: Put the instruments over night in a saturated solution of zinc chloride. After the spots have disappeared the instruments should be immersed in hot soda-lye and then carefully wiped dry. A further cleansing with pure alcohol and whiting is also recommended. Rust-spots may also be removed with petroleum.

In order to protect instruments against rust, the *Pharm. Centralblatt*, No. 28, recommends the use of paraffin oil in the following manner: First dry the instruments thoroughly in warm air, and then dip them in a solution of 1 part of paraffin-oil to 200 parts of benzine. Instruments like forceps and scissors should be worked beneath the surface, so that the fluid can penetrate thoroughly into the grooves. The instruments should then be laid upon a board in a dry room so that the benzine can evaporate.

Suture-needles can be merely immersed in the paraffin solution and kept in a diagonal or slanting direction, and then laid on a board to dry. Too much paraffin-oil will not then be taken up by the instrument.—*Idem.*

